# Generation of dyskeratosis congenita-like hematopoietic stem cells through the stable inhibition of *DKC1*

**DOI:** 10.1186/s13287-021-02145-8

**Published:** 2021-01-29

**Authors:** Carlos Carrascoso-Rubio, Hidde A. Zittersteijn, Laura Pintado-Berninches, Beatriz Fernández-Varas, M. Luz Lozano, Cristina Manguan-Garcia, Leandro Sastre, Juan A. Bueren, Rosario Perona, Guillermo Guenechea

**Affiliations:** 1 Instituto de Investigaciones Biomédicas Alberto Sols (CSIC/UAM), 28029 Madrid, Spain; 2 Division of Hematopoietic Innovative Therapies, Centro de Investigaciones Energéticas Medioambientales y Tecnológicas (CIEMAT), Avenida Complutense, 40, 28040 Madrid, Spain; 3 Centro de Investigación Biomédica en Red de Enfermedades Raras (CIBERER), 28029 Madrid, Spain; 4 Advanced Therapies Unit, Instituto de Investigación Sanitaria Fundación Jiménez Díaz (IIS-FJD/UAM), 28040 Madrid, Spain

**Keywords:** Dyskeratosis congenita, *DKC1* gene, Bone marrow failure disorders, Hematopoietic stem cells, Short hairpin RNA, Lentiviral vectors

## Abstract

Dyskeratosis congenita (DC) is a rare telomere biology disorder, which results in different clinical manifestations, including severe bone marrow failure. To date, the only curative treatment for the bone marrow failure in DC patients is allogeneic hematopoietic stem cell transplantation. However, due to the toxicity associated to this treatment, improved therapies are recommended for DC patients. Here, we aimed at generating DC-like human hematopoietic stem cells in which the efficacy of innovative therapies could be investigated. Because X-linked DC is the most frequent form of the disease and is associated with an impaired expression of *DKC1*, we have generated DC-like hematopoietic stem cells based on the stable knock-down of *DKC1* in human CD34^+^ cells with lentiviral vectors encoding for *DKC1* short hairpin RNAs. At a molecular level, *DKC1*-interfered CD34^+^ cells showed a decreased expression of *TERC*, as well as a diminished telomerase activity and increased DNA damage, cell senescence, and apoptosis. Moreover, *DKC1*-interfered human CD34^+^ cells showed defective clonogenic ability and were incapable of repopulating the hematopoiesis of immunodeficient NSG mice. The development of DC-like hematopoietic stem cells will facilitate the understanding of the molecular and cellular basis of this inherited bone marrow failure syndrome and will serve as a platform to evaluate the efficacy of new hematopoietic therapies for DC.

**Supplementary Information:**

The online version contains supplementary material available at 10.1186/s13287-021-02145-8.

## Introduction

Telomeres are repetitive nucleotide sequences localized at the end of the eukaryotic chromosomes, which play an essential role in the chromosome replication and stability. Telomeric DNA consists of tandemly repeated TTAGGG sequences [1, 2] which become shortened as a consequence of the division of somatic cells, leading to a situation called “end replication problem”. The loss of telomeric repeats is counteracted by the telomerase complex [3]. Telomerase is a specialized ribonucleoprotein reverse transcriptase mainly composed of TERT (with reverse transcriptase activity), TERC (the RNA template) and dyskerin, which stabilizes telomerase complex [4–6]. Although telomerase expression is low or absent in most somatic cells, telomerase remains active in somatic stem cells to maintain their telomere length [7]. A decreased telomerase activity results in an abnormal telomere biology, leading to telomere biology disorders (TBD), such as aplastic anemia, pulmonary fibrosis, coats plus syndrome, or dyskeratosis congenita (DC) [2, 8].

Clinically, DC patients are characterized by the mucocutaneous triad (nail dystrophy, oral leukoplakia, and abnormal skin pigmentation). Nevertheless, bone marrow failure (BMF) is the main cause of early mortality of these patients (80% of the cases) as also occurs in other congenic BMF syndromes [7]. So far, 14 DC associated genes have been discovered, all of them involved in the telomere maintenance: *DKC1*, *TERT*, *TERC*, *TINF2*, *TCAB1*, *NOP10*, *NHP2*, *CTC1*, *RTEL1*, *TPP1*, *PARN*, *POT1*, *NAF1*, and *STN1* [9–12]. According to the inheritance of the disease, three DC variants have been reported: X-linked recessive, autosomal dominant, and autosomal recessive. The X-linked variant of DC (X-DC) is mainly caused by point mutations in *DKC1*, which encodes for the dyskerin nucleolar protein [13]. Interestingly, the knock-out of *Dkc1* has been reported to be embryonic lethal in mice [14]. This observation and the fact that only hypomorphic *DKC1* mutations have been reported in X-DC patients [15, 16] reveals the critical relevance of *DKC1* in the cell biology.

To date, the only curative treatment for BMF in DC patients is the allogeneic hematopoietic stem cell transplantation (alloHSCT) from healthy donors. Apart from the low availability of HLA-matched donors, the outcome of DC patients undergoing alloHSCT is very poor, mainly due to the toxicity of conditioning regimens and the development of graft versus host disease [17]. Thus, new therapies such as gene therapy without cytotoxic conditioning, as recently reported in Fanconi anemia (FA) [18], would be highly beneficial for DC patients.

Taking into account that periodic BM aspirations are not part of the routine follow-up of DC patients, difficulties in the access of HSCs constitute an important limitation in the development of new therapies for DC patients. Furthermore, the animal models of telomeropathies developed to date do not mimic the characteristic BMF of DC patients [19]. Considering that *DKC1* is one the most frequently mutated genes in DC [9], the purpose of this study was the generation of DC-like human HSCs based on the interference of *DKC1* in human HSCs which would serve as a platform for the development of new hematopoietic therapies for DC patients.

## Materials and methods

Detailed methods are shown as supplementary data

## Results

### Molecular implications of *DKC1* inhibition in human hematopoietic stem and progenitor cells

Previous studies revealed that the knock-out of *Dkc1* is embryonic lethal [14] and that only hypomorphic mutations have been found in X-DC patients [15, 16]. In this study, we aimed at generating X-DC-like hematopoietic stem and progenitor cells (HSPCs) based on the downregulation of *DKC1* with short hairpin RNA (shRNA) lentiviral vectors (LVs). shRNA-LVs carried a puromycin resistance gene to facilitate the selection of transduced HSPCs (see the “Materials and methods” section).

**Fig. 1:**
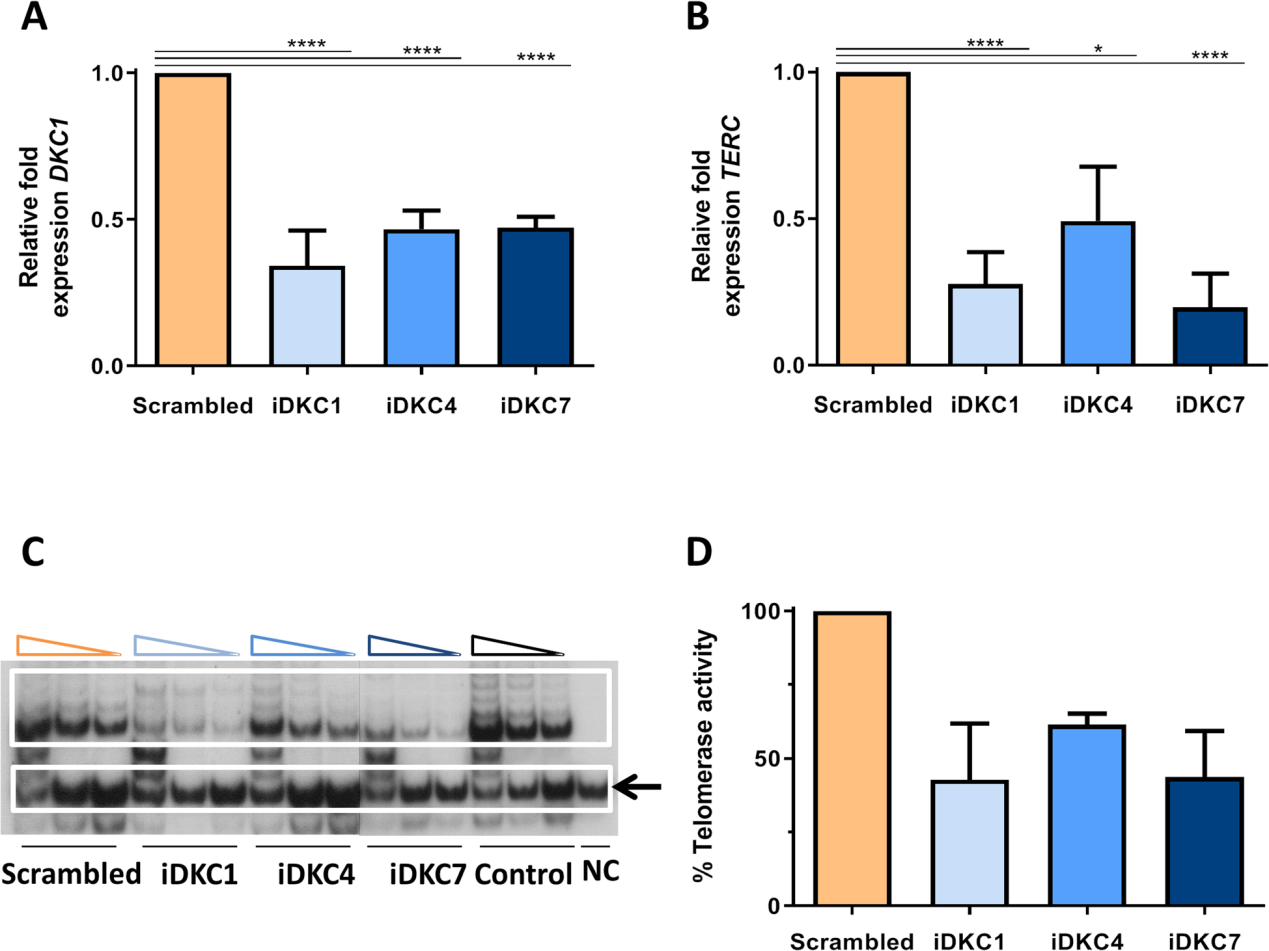
Molecular implications associated with the inhibition of *DKC1* in human hematopoietic stem and progenitor cells. Cord blood CD34^+^ cells were transduced with specific anti shRNA-LVs and maintained in liquid cultures for 5–8 days (see details in the “Materials and methods” section). **a** Decreased expression of *DKC1* gene after CD34^+^ cell transduction with specific shRNA-LVs (7 independent experiments were conducted; *n* = 7). *DKC1* expression levels in cells transduced with *DKC1* shRNA LVs represent relative values of those obtained in cells transduced with the scrambled shRNA LV. Raw data are shown in Suppl. Table 2. **b** Decreased expression of *TERC* after CD34^+^ cell transduction with specific shRNA-LVs (*n* = 7). Raw data are shown in Suppl. Table 2. **c** Representative analysis of a telomeric repeat amplification protocol (TRAP). Internal control is marked by the black arrow, negative control was performed with buffer (NC) and non-transduced cells were used as control. **d** Analysis of the telomerase activity after transduction with shRNA LVs (*n* = 3). Data are expressed as mean ± SEM. Asterisks indicate significant differences determined by Student’s *t* test (*p < 0.05, **p < 0.01, ***p < 0.001, ****p < 0.0001)

The efficacy of seven different shRNA-LVs (Suppl. Table 1) to downregulate the expression of *DKC1* was screened in healthy donor CD34^+^ cells (Suppl. Fig. 1A). In subsequent experiments, we showed that three of these shRNA-LVs, iDKC1, iDKC4, and iDKC7 significantly decreased *DKC1* mRNA levels to 34–47% compared to levels determined in cells transduced with the scrambled shRNA LV (Fig. 1a and Suppl. Table 2A). Vector copy numbers (VCN) determined in these cells showed the presence of 1–8 copies per cell in all groups (Suppl. Fig. 1B), revealing that inhibitory effects upon *DKC1* were related to the interfering proviruses.

To investigate the molecular implications resulting from the inhibition of *DKC1*, we first evaluated the expression of *TERC* in CD34^+^ cells transduced with scrambled and *DKC1*-shRNA LVs. As shown in Fig. 1b and Suppl. Table 2B, *TERC* mRNA levels in cells transduced with iDKC1-, iDKC4-, or iDKC7-LVs were respectively decreased to 27.7% ± 10.8%, 49.1% ± 18.6%, and 19.8% ± 11.5%, compared to levels determined in the control group. In subsequent analyses, changes in the telomerase functionality of *DKC1*-interfered CD34^+^ cells were quantified. To this end, we measured telomerase activity of *DKC1-*interfered and control CD34^+^ cells by the TRAP assay. These results showed marked decreases in the telomerase activity of CD34^+^ cells that had been transduced with iDKC1-, iDKC4-, or iDKC7-LVs, which showed values of 42.8% ± 19%, 61.5% ± 3.6%, and 43.7% ± 15.6%, respectively, of values determined in the control group (Fig. 1c and d).

**Fig. 2:**
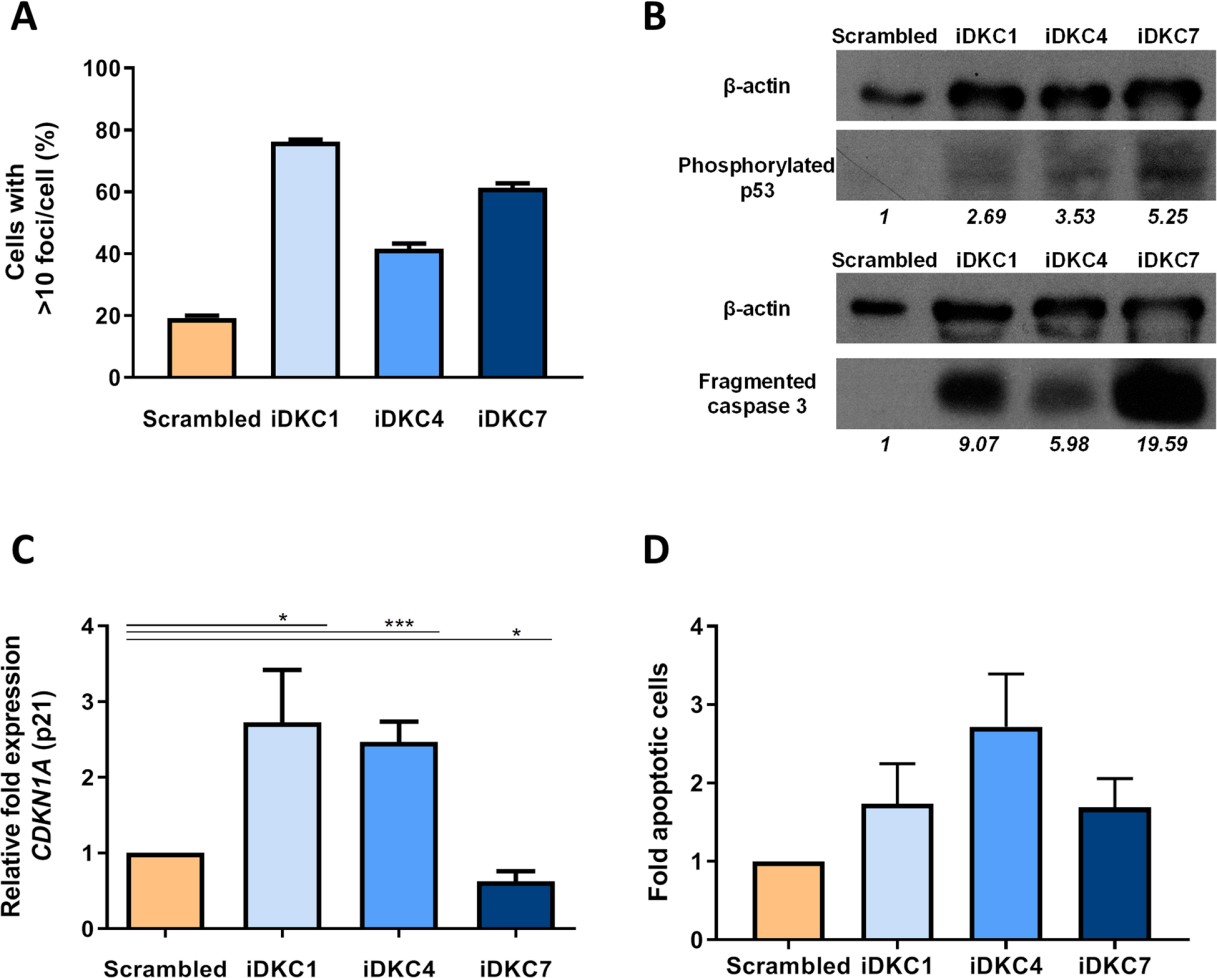
DNA damage and apoptosis associated with the inhibition of *DKC1* in human hematopoietic stem and progenitor cells. Cord blood CD34^+^ cells were transduced with specific anti-shRNA-LVs and maintained in liquid cultures for 10 days (see details in “Materials and methods” section). **a** Analysis of DNA damage in CD34^+^ cells transduced with specific shRNA LVs. Cells with more than 10 γH2AX foci per cell are shown (*n* = 3). **b** Representative Western blot (WB) assays for phosphorylated p53 (upper WB) and fragmented caspase 3 (lower WB) expression using β-actin as control. Quantification appears in italics below the images as the ratio of expression in relation with the control protein. **c** Increased expression of p21 after transduction of CD34^+^ cells with specific shRNA-LVs (*n* = 6). Raw data are shown in Suppl. Table 2. **d** Fold increase of apoptotic cells (Annexin V^+^) in comparison with the scrambled control condition (*n* = 3). Data are expressed as mean ± SEM. Asterisks indicate significant differences determined by Student’s *t* test (*p < 0.05, **p < 0.01, ***p < 0.001, ****p < 0.0001)

In the following experiments, we investigated the implication of *DKC1* interference in the DNA damage determined in CD34^+^ cells. Analyses of γH2AX foci in the nucleus of cells transduced with iDKC1-, iDKC4-, or iDKC7-LVs revealed that only 19% of cells transduced with the scrambled shRNA LV showed more than 10 γH2AX foci per cell. However, an important increase in the proportion of CD34^+^ cells with γH2AX foci was observed in cells transduced with either the iDKC1- (76%), iDKC4- (42%), or the iDKC7- (61%) LVs (Fig. 2a). In next studies, we determined the expression of phosphorylated p53 and p21 (*CDKN1A*) in CD34^+^ cells transduced with the different constructs. As shown in Fig. 2b, phosphorylated p53 expression was higher in CD34^+^ cells transduced with the *DKC1*-shRNA LVs. When the expression of p21 was tested, iDKC1- and iDKC4-LVs enhanced its levels (2.7 ± 0.7 and 2.4 ± 0.26 fold, respectively) compared to the control group, though this was not observed in iDKC7-transduced cells (Fig. 2c and Suppl. Table 2C). Levels of caspase 3 and Annexin V^+^ cells were also increased in CD34^+^ cells transduced with either type of *DKC1*-shRNA LVs, although levels did not reach statistical significance (Fig. 2b and d and Suppl. Fig. 2). Taken together these results suggest the induction of DNA damage, cell senescence, and apoptosis of *DKC1*-interfered HSPCs (Fig. 2).

### The interfered expression of *DKC1* impairs the in vitro growth and ablates the in vivo repopulating ability of human HSPC

**Fig. 3:**
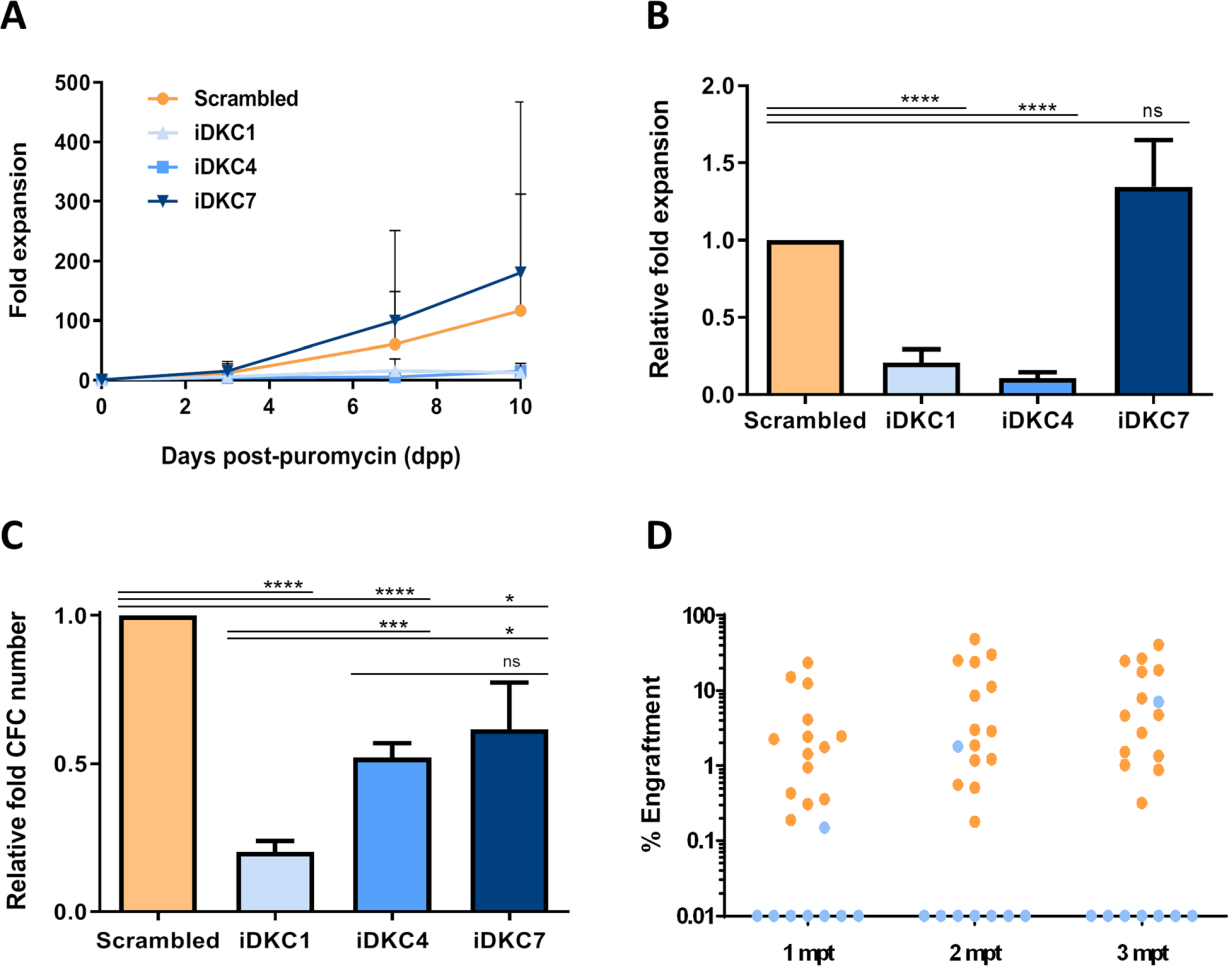
Analysis of the in vitro growth properties and in vivo repopulating ability of *DKC1*-interfered CD34^+^ cells. **a** Analysis of the cell expansion analyzed 2 weeks after ex vivo incubation of transduced cells in liquid culture (*n* = 5). **b** Relative cell expansion in comparison with cells transduced with the scrambled shRNA-LV (*n* = 6). **c** Analysis of the clonogenic potential of CD34^+^ cells transduced with *DKC1*-shRNA LVs and scrambled shRNA-LVs (*n* = 8). **d** Analysis of the repopulation potential of CD34^+^ cells transduced with scrambled shRNA-LV (orange dots) or *DKC1*-shRNA LVs (blue dots). The proportion of human CD45^+^ cells in the BM of recipient mice was analyzed at 1–3 months post-transplantation (mpt). Data are expressed as mean ± SEM. Asterisks indicate significant differences determined by Student’s *t* test (*p < 0.05, **p < 0.01, ***p < 0.001, ****p < 0.0001)

To determine whether the knockdown of *DKC1* affects the functionality of human HSPCs, *DKC1*-interfered CD34^+^ cells were in vitro cultured for 10 days (see the “Materials and methods” section) to evaluate implications in cell growth. In these studies, the portion of CD34^+^ cells at the end of the culture period was similar among the different experimental groups (Suppl. Fig. 3**)**. While transduced cells with the scrambled shRNA-LV showed a marked cell expansion during this period (117 ± 87.31 fold compared to initial cell numbers), levels of expansion observed in iDKC1- and iDKC4-transduced CD34^+^ cells were only 13 ± 6.99 and 15.3 ± 2.42 fold compared to input cell numbers (Fig. 3a). These values represent a significant decrease to 20 ± 8% and 10 ± 4%, respectively, of cell expansions corresponding to the control group (CD34^+^ cells transduced with the scrambled shRNA LV) (Fig. 3b). As happened with p21 levels (Fig. 2c), defects in cell proliferation were not observed with iDKC7-transduced cells (Fig. 3b). In additional studies, we evaluated changes in the telomere length in *DKC1*-interfered cells, although no differences were observed among the different experimental groups (Suppl. Fig. 4). This suggests that much longer incubation periods would be required to observe a significant telomere shortening, although defects in the ability of *DKC1*-interfered cells to grow in culture limited the possibility of evaluating changes in the telomere length long-term after *DKC1*-interference.

When the clonogenic potential of *DKC1-*interfered cells was assessed, a significant reduction in the number of colonies generated by CD34^+^ cells transduced with any of the three anti-*DKC1* LVs was observed (Fig. 3c and Suppl. Fig. 5). Again, reductions were more significant in cells transduced with the iDKC1- and iDKC4-LVs, which reduced the clonogenic potential to 20% ± 4% and 52% ± 5%, respectively, compared to the control group.

Based on the results obtained in cells transduced with iDKC1-LV, in a final set of experiments, we assessed the repopulation potential of CD34^+^ cells transduced with this LV, and with a control LV (scrambled shRNA-LV). To this aim, 8 × 10^5^ transduced cells, which contained an average number of 30,000 transduced CD34^+^ cells (Suppl. Fig. 3), were transplanted into NSG mice. As shown in Fig. 3d, CD34^+^ cells transduced with the control LV showed an evident in vivo repopulating ability (see orange dots in Fig. 3d). In these animals, the presence of human hematopoietic progenitors (CD34^+^), as well as of myeloid (CD33^+^) and lymphoid cells (CD19^+^) were observed (Suppl. Fig. 6), confirming the multi-lineage repopulation ability of human HSPCs transduced with the scrambled shRNA LVs. In sharp contrast with these observations, 7 out of the 8 recipients that were transplanted with iDKC1-transduced CD34^+^ cells failed to repopulate recipient NSG mice (see light blue dots in Fig. 3d). Interestingly, when VCNs were tested in the BM of mice engrafted with cells of the control group, the presence of integrated LV copies was observed in all cases (0.3 to 1 VCNs/cell; Suppl. Fig. 7). However, no copies of the iDKC1 provirus were detected in BM cells from the animal engrafted with cells transduced with the *DKC1*-shRNA LV (Suppl. Fig. 7). This reveals that this specific recipient was repopulated with cells that have survived the puromycin selection, although did not integrate in their genome the iDKC1-interfering provirus. As expected, the presence of the *DKC1*-shRNA provirus was neither observed in the non-engrafted NSG recipients (Suppl. Fig. 7), since no human hematopoietic cells were observed in these recipients (Fig. 3d).

Based on the hematopoietic studies conducted in these experiments, we conclude that the inhibited expression of *DKC1* impairs the in vitro growth properties and the in vivo repopulating ability of human HSPCs.

## Discussion

The absence of good models which mimic HSC defects characteristic of DC patients [20] constitute an important limitation in the development of therapies for the treatment of BMF of these patients [21]. In this study, we show that three different *DKC1*-shRNAs inhibited *DKC1* expression to levels below 50%, similar to observations in X-DC patients, all of them with hypomorphic mutations in *DKC1* [22, 23]. Consistent with data from these patients [22–25], *DKC1* inhibition in healthy HSPCs was associated with a significant reduction in the expression of *TERC* and of telomerase activity. As also observed in cells from DC patients, *DKC1* interference with iDKC1- and iDKC4-LVs induced markers of DNA damage, cell senescence, and apoptosis, such as the generation of nuclear γH2AX foci and upregulation of caspase 3, p21, and phosphorylated p53.

Consistent with observations showing that BM from DC patients contain reduced numbers of HSPCs [26], *DKC1* interference with iDKC1- and iDKC4-LVs markedly reduced the cell expansion, as well as the clonogenic and in vivo repopulating potential of CD34^+^ cells. The fact that, in contrast to iDKC1- and iDKC4-, iDKC7-LV did not increase levels of p21 nor affected the cell growth of CD34^+^ cells suggests the different functional implications associated with the interference of different domains of *DKC1*.

Remarkably, defects in the in vitro and in vivo growth of human HSPCs were evident immediately after *DKC1* interference, despite no changes in the telomere length of these cells were observed. This observation indicates that the inhibited proliferation and repopulation ability of DC-like HSPCs, and most probably of HSCs from X-DC patients, are not necessarily a consequence of the reduced telomere length. Thus, we propose that the generation of DNA damage and induction of cell senescence and apoptotic responses would account for these relevant phenotypic defects of DC HSPCs. Although the inability of *DKC1*-interfered HSCs to engraft in immunodeficient mice would limit studies of the behavior of these cells in vivo, this model will be an invaluable tool to evaluate the efficacy of ex vivo therapies, such as hematopoietic gene therapy, to restore the repopulating properties of HSCs defective in *DKC1*. Moreover, the repopulation defects observed in our study in DC-like HSPCs would suggest that the restored function of dyskerin through gene therapy strategies might confer a proliferation advantage in DC HSPCs, as we have already demonstrated in FA patients treated by hematopoietic gene therapy [27].

Aiming at restoring the function of X-DC cells, discrepant results have been observed after the ectopic expression of dyskerin [22, 28, 29]. The use of codon optimized sequences of *DKC1* (not recognized by *DKC1*-shRNAs) or the use of functionally active *DKC1*-derived sequences, such as those encoding for GSE24.2 and GSE4 peptides [29–31], might compensate the molecular and cellular defects of DC HSCs. As proposed for FA [27], the correction of HSCs in early stages of the disease of DC would be also relevant to complement the function of affected genes before telomeres are significantly reduced. Whether or not gene complementation in DC HSPCs with shortened telomeres would facilitate their elongation is currently unknown and will require extensive studies in this and other DC models.

## Conclusion

The generation of DC-like HSPCs constitutes a new platform for studying the molecular basis of the BMF in DC and also for screening the efficacy and safety of hematopoietic therapies for DC patients, including gene therapy and drugs capable of protecting or restoring the function of DC HSPCs.

## Supplementary Information

**Additional file 1: ** Supplementary Table 1. Overview of the information about the shRNA sequences. Supplementary Table 2. Compilation of RT-qPCR data in CD34^+^ transduced with scrambled and *DKC1*-shRNA LVs**. A**) Results of *DKC1* expression (n = 7). B) *TERC* expression data (n = 7). **C**) Expression of *CDKN1A* gene (n = 6). Triplicates were performed in every experiment and results are expressed as mean ± SEM

**Additional file 2: **Supplementary Figure 1. A) *DKC1* expression after transduction with a library of *DKC1*-specific shRNAs, n=3. Analyses were performed 5-8 days post-transduction. B) Analysis of the vector copy number per cell (VCN/cell) after transduction with shRNA-LVs. Analyses were performed at least after 15 days post-transduction. Supplementary Figure 2. Gating strategy and analysis of Annexin V-based apoptosis assay. Representative dot-plots of flow cytometry analyses in *DKC1*-interferred CD34^+^ cells are shown. Supplementary Figure 3. Levels of CD34^+^ cells after 10 days in culture (n=3). Supplementary Figure 4. Telomere length in human CD34^+^ samples transduced with *DKC1-shRNAs* and scrambled-shRNA LVs and cultured for 8 days as described in Materials and Methods section. For comparison, also human peripheral blood cells and fresh CD34^+^ cells were analyzed (n=1). Supplementary Figure 5. Analysis of the clonogenic potential, discriminating between GM-CFU (A) and E-BFU (B) colonies, of CD34^+^ cells transduced with *DKC1*-shRNA LVs and scrambled shRNA-LVs (n=8). Data are expressed as mean ± SEM. Asterisks indicate significant differences determined by Student’s t test (*p < 0.05, **p < 0.01, ***p < 0.001, ****p < 0.0001). Supplementary Figure 6. Gating strategy and analysis of the NSG mice repopulating potential of CD34^+^ cells transduced with iDKC1 and scrambled-shRNA LVs. Representative dot-plots of flow cytometry analyses performed in the bone marrow samples from two mice transplanted with scrambled-shRNA LVs are shown. Supplementary Figure 7. Analysis of the VCNs in the bone marrow of recipient NSG mice at 3 months post-transplantation. NSG mice were transplanted with scrambled transduced CD34^+^ cells (orange dots, n=14) or iDKC1 interfered CD34^+^ cells (blue dots, n=8)

## Data Availability

The authors confirm that the data supporting the findings of this study are available from the corresponding author on reasonable request.

## Abbreviations

AlloHSCT: Allogeneic hematopoietic stem cell transplantation
BM: Bone marrow
BMF: Bone marrow failure
DC: Dyskeratosis congenita
FA: Fanconi anemia
HSC: Hematopoietic stem cell
HSPC: Hematopoietic stem and progenitor cell
LV: Lentiviral vector
shRNA: Short hairpin RNA
TBD: Telomere biology disorder
TERC: Telomerase RNA component
TERT: Telomerase reverse transcriptase
TRAP: Telomeric repeat amplification protocol
VCN: Vector copy number
WB: Western blot
X-DC: X-linked dyskeratosis congenita
