# Supplementary material for: Generation of dyskeratosis congenita-like hematopoietic stem cells through the stable inhibition of DKC1
Source: Stem Cell Res Ther. 2021 Jan 29;12:92. doi: 10.1186/s13287-021-02145-8 (PMC7844988; doi:10.1186/s13287-021-02145-8)
Supplement: Supplementary file 1 — Additional file 1: Supplementary Table 1. Overview of the information about the shRNA sequences. Supplementary Table 2. Compilation of RT-qPCR data in CD34+ transduced with scrambled and DKC1-shRNA LVs. A) Results of DKC1 expression (n = 7). B) TERC expression data (n = 7). C) Expression of CDKN1A gene (n = 6). Triplicates were performed in every experiment and results are expressed as mean ± SEM [file 13287_2021_2145_MOESM1_ESM.docx]

**List of abbreviations**

BM: Bone marrow

CB: Cord blood

CFC: Colony forming cell

E-BFU: Erythroid-burst forming unit

GM-CFU: Granulomacrophagic-colony forming unit

LV: Lentiviral vector

mpt: Months post-transplant

NSG: NOD-SCID-Il2rg^-/-^ mouse strain

qPCR: Quantitative polymerase chain reaction

qRT-PCR: Quantitative reverse transcription polymerase chain reaction

SEM: Standard error of mean

shRNA: Short hairpin RNA

TRAP: Telomeric repeat amplification protocol

VCN: Vector copy number

**Materials and Methods**

***Human hematopoietic cells***

Mononuclear cells from pooled umbilical cord blood samples (CB) were obtained through a density gradient using Ficoll-Paque (GE Healthcare) and CD34^+^ cells were purified using the MACS CD34 Micro-Bead kit (Miltenyi Biotec). Umbilical CB samples were kindly provided by the *Centro de Transfusión de la Comunidad de Madrid*. In all instances informed consents were previously signed from the mothers.

Cells were cultured in StemSpam (StemCell Technologies) medium supplemented with 1% GlutaMAX (Gibco), 1% penicillin/streptomycin solution (Gibco), 100 ng/ml hTPO, hFIT3L and hSCF and 20 ng/ml IL-3 (all growth factors from EuroBiosciences).

***Lentiviral vectors and generation of human X-DC-like HSPCS***

Second-generation self-inactivating lentiviral vectors (LVs) were generated in HEK 293T cells (ATCC). These cells were grown in Dulbecco’s modified Eagle’s medium (DMEM, Gibco) supplemented with 10% fetal bovine serum (FBS, Sigma-Aldrich) and 0.5% penicillin/streptomycin (P/S, Gibco). Supernatants were collected at 24 and 48 hours post-transfection and viruses were concentrated by centrifugation at 50000g for 2 hours. Titers were determined by flow cytometry or quantitative polymerase chain reaction (qPCR) in 293T cells, plated at 5x10^4^ cells per well in 24-well plates and infected with serial dilutions of LVs overnight, after 14 days of culture.

LVs titration was performed in HEK 293T cultured in DMEM (Gibco) supplemented as previously described. Cells were transduced with serial dilutions of the corresponding LV. When the LV carried the EGFP marker, cells were analyzed by flow cytometry after 5-12 days of liquid culture. In the remaining cases, titration was conducted by qPCR in an Applied 7500 Fast Real Time PCR system (Thermo Fisher Scientific).

Seven short hairpin RNAs (shRNAs) were respectively inserted into the pLKO.1 LV (GE Healthcare Life Sciences), which also carry the puromycin resistance gene (**Suppl.** **Table 1**). A pGIPZ LV with a scrambled shRNA sequence was used as control. To generate DC-like HSPCS, healthy CD34^+^ cells were transduced with these LVs at a MOI of 100 IU/cell as previously described (1). Two cycles of transduction were performed to increase the percentage of transduction and the vector copy number (VCN). In all instances, transduced CD34^+^ cells with LVs-encoding the different shRNAs were selected for 72 h in liquid cultures containing 1 µg/ml of puromycin to prevent the generation of untransduced CD34^+^ cells. Under these conditions no hematopoietic colonies were generated by untransduced samples.

Membrane expression of CD34 antigen was analyzed by flow cytometry using hCD34 (555824, Becton Dickinson) monoclonal antibody according to the manufacturer’s instructions. Samples were analyzed on the LSR Fortessa (BD Biosciences) and results were processed with FlowJo V10 software (FlowJo, LLC).

***Analysis of lentiviral vector copy number***

When CD34^+^ cells were analyzed, samples were maintained *in vitro* for 2 weeks to prevent the analysis of non-integrated copies of the LV. DNA was extracted using the DNeasy Blood & Tissue kit (Qiagen). Genomic DNA from hematopoietic colonies was obtained by adding 20 mL of lysis buffer (0.3 mM Tris HCl, pH 7.5; 0.6 mM CaCl_2_; 1.5% Glycerol; 0.675% Tween-20; and 0.3 mg/mL Proteinase K). Lysis protocol consists on 30 minutes at 65ºC, 10 minutes at 90ºC and 10 minutes at 4ºC. LV vector copy number (VCN) was quantified by qPCR detection of *Psi* sequence (specific primers for *Psi* were *Psi* forward (Fw) 5’-CAGGACTCGGCTTGCTGAAG-3’ and *Psi* reverse (Rv) 5’-TCCCCCGCTTAATACTGACG-3’ with a Taqman probe *Psi* FAM 5’-CGCACGGCAAGAGGCGAGG-3’) normalized to endogenous human albumin gene (h*Alb*) (specific primers for h*Alb* were *Alb* Fw 5’-GCTGTCATCTCTTGTGGGCTG-3’ and *Alb* Rv 5’-ACTCATGGGAGCTGCTGGTTC-3’ with a Taqman probe *Alb* VIC 5’-CCTGTCATGCCCACACAAATCTCTCC-3’). qPCR was performed in an Applied 7500 Fast Real Time PCR system (Thermo Fisher Scientific), as previously described (2).

***Gene expression assays***

*DKC1-*interfered CD34^+^ cells were pelleted and RNA was extracted using TRIzol Reagent (Invitrogen). The expression of *DKC1*, *TERC* and *CDKN1A* mRNA was analyzed by real-time quantitative reverse transcriptase-PCR (qRT-PCR) of cDNA obtained from total RNA from *DKC1-*interfered CD34^+^ cells. Untransduced samples and samples transduced with scrambled LV were used as negative controls.

The relative expression of *DKC1*, *TERC* and p21 (*CDKN1A*) was determined in a StepOne Plus Real-Time PCR System (Applied Biosystems) by the 2^–ΔΔCt^ method as previously described (3), using Power SYBR Green kit (Applied Biosystems) and the following primers: *DKC1* forward (Fw) 5’-GCTCCTCAGTTGATCAAGAAGG-3’ and reverse (Rv) 5’-CTCAGAAAACCAATTCTACCTC-3’, *TERC* Fw 5’-TCTAACCCTAACTGAGAAGGGCGTAG-3’ and Rv 5’ GTTTGCTCTAGAATGAACGGTGGAAG-3’ and *CDKN1A* (p21) Fw 5’-GCTGCAGGGGACAGCAGAG-3’ and Rv 5’-GCTTCCTCTTGGAGAAGATCAG-3’. For housekeeping control expression human glyceraldehyde-3-phosphate dehydrogenase (*GAPDH*) was used Fw 5’-GAGAGACCCTCACTGCTG-3’ and Rv 5’-GATGGTACATGACAAGGTGG-3’.

***Telomeric repeat amplification protocol assay***

Telomerase activity was determined under recommended standard conditions of the TRAPEZE Telomerase Detection S7700 Kit (Millipore) for telomeric repeat amplification protocol (TRAP) using radioisotopic detection. Telomerase activity was determined in each sample using 0.5, 0.25 and 0.125 μg of protein extract and normalized with the internal control included in the assay (4, 5).

***Analysis of DNA damage***

CD34^+^ cells were maintained 24 hours in 4-chambers Falcon CultureSlides (Corning) pre-coated with 5 µg/cm2 of Retronectin (Takara). DNA damage was analyzed by determining and quantifying histone γH2AX foci. The γH2AX localization was carried out by fluorescence microscopy. For this purpose, cells were fixed in 3.7% formaldehyde solution (Fluka, Sigma-Aldrich) at room temperature for 15 min. After washing with 1x PBS, cells were permeabilized with 0.2% Triton X-100 in PBS and blocked with 10% horse serum (Sigma-Aldrich) before overnight incubation with γH2AX antibody. Finally, cells were washed and incubated with secondary antibodies coupled to fluorescent dyes (Alexa Fluor 488 or/and Alexa Fluor 647). Images were acquired with a Confocal Spectral Leica TCS SP5 using a HCX PL APO lambda blue 63x/1.4 oil UV, zoom 2.3 lens. Images were acquired using LAS-AF 1.8.1 Leica software and processed using LAS-AF 1.8.1 Leica software and Adobe Photoshop CS. At least 200 cells were evaluated for γH2A.X staining.

***Western blot for phosphorylated*** ***p53***

Whole cell extracts from CD34^+^ were prepared using lysis buffer (0.3 M NaCl, 25 mM HEPES pH 7.5, 20 mM β-glycerophosphate, 1.5 mM MgCl_2_, 0.2 mM EDTA, and 0.1% Triton X-100) and a protease inhibitor cocktail (0.5 mM AEBSF, 2 µg/ml Leupeptin, 2 µg/ml Aprotinin, 0.5 mM DTT and 0.1 mM Na_3_VO_4_). Lysates were centrifuged and protein concentration was measured by using the Bio-Rad Protein Assay (500-0006, Bio-Rad). 20 µg of protein extract were resolved on SDS-polyacrylamide and electroblotted to Immobilon P membranes (Merck Millipore). Western blot analysis was conducted by using ECL reagent (Santa Cruz Biotechnology).

The source of antibodies was as follows: phospho-p53 (Ser 15) (9284, Cell Signaling), caspase-3 (9662, Cell Signaling) and β-actin (C4) (sc-47778, Santa Cruz Biotechnology).

***Analysis of apoptosis***

After 10 days in culture, apoptosis of *DKC1*-interfered CD34^+^ cells was studied. Cells were resuspended in 100 µl Annexin Binding Buffer 1x (556454, BD Pharmingen) then. A mix composed of 10 µl of PBA, 2 µl DAPI (D9542, Sigma-Aldrich) and 2 µl PE Annexin V (556422, BD Pharmingen) was added to each sample and incubated at room temperature in the dark for 15 minutes. After that, 300 µl Annexin Binding Buffer 1x was added and samples were analyzed on the LSR Fortessa (BD Biosciences). Results were processed with FlowJo V10 software (FlowJo, LLC).

***Colony-Forming Cell Assays***

To determine the number of colony-forming cells (CFCs), *DKC1-*interfered CD34^+^ cells were cultured in 35 mm plates in methylcellulose medium (Methocult) for 14 days at 37ºC, 5% CO_2_ and 90% relative humidity. After that time, granulocyte-macrophage colony-forming units (GM-CFU) and erythroid burst-forming units (E-BFU) were quantified using an inverted microscope (Nikon).

***Telomere length***

The relative telomere length was assessed by quantitative Polymerase Chain Reaction (qPCR) as previously described (6). The qPCR determines the ratio of telomere (T) repeat copy number to single-copy (S) gene (36B4) copy number (T/S ratio) in experimental samples, as compared with a reference DNA sample (MCF7 cells).

***Hematopoietic Reconstitution potential***

NOD-SCID-Il2rg^-/-^ (NSG) mice were obtained from the Jackson Laboratory and maintained at the CIEMAT animal facilities (registration number 28079-21 A). All the experimental procedures were approved by the Animal Welfare Body of the CIEMAT and the project was authorized by the competent authorities of the *Comunidad de Madrid*, under the registration number PROEX-70/15, and thus fulfilling Spanish and European legislation (Spanish RD 53/2013 and Law 6/2013 in compliance with the European Directive 2010/63/EU about the use and protection of vertebrate mammals used for experimentation and other scientific purposes). NSG mice were previously irradiated with 1.5 Gy (X-rays; MG324 Philips) 24 hours before intravenous HSC transplantation through retro-orbital sinus. Human hematopoietic engraftments were evaluated by performing femoral bone marrow (BM) aspirations at 1 and 2 months post-transplant (mpt). After 3 mpt, transplanted mice were culled and femoral BM cells were obtained. Samples were analyzed by flow cytometry using hCD45 (304014, BioLegend), hCD33 (A07775, Beckman Coulter), hCD19 (25-0198-42, eBioscience) and hCD34 (555824, Becton Dickinson) monoclonal antibodies according to the manufacturer’s instructions. All these analysis were performed on the LSR Fortessa (BD Biosciences) and processed with FlowJo V10 software (FlowJo, LLC).

***Statistics***

All statistical analyses were performed using GraphPad Prism 8.00. Results are reported as mean ± standard error of mean (SEM) and significance was set as p ≤ 0.05 throughout the experiments. Statistical differences between means were evaluated by Student’s t test after checking the Gaussian distribution of the sampled data using Shapiro-Wilk normality test. For nonparametric variables (n < 5), statistical evaluation was performed through Mann-Whitney test.

**References**

1. Rio P, Navarro S, Guenechea G, Sanchez-Dominguez R, Lamana ML, Yanez R, et al. Engraftment and in vivo proliferation advantage of gene-corrected mobilized CD34(+) cells from Fanconi anemia patients. Blood. 2017 Sep 28;130(13):1535-42. PubMed PMID: 28801449.

2. Gonzalez-Murillo A, Lozano ML, Alvarez L, Jacome A, Almarza E, Navarro S, et al. Development of lentiviral vectors with optimized transcriptional activity for the gene therapy of patients with Fanconi anemia. Hum Gene Ther. 2010 May;21(5):623-30. PubMed PMID: 20001454.

3. Livak KJ, Schmittgen TD. Analysis of relative gene expression data using real-time quantitative PCR and the 2(-Delta Delta C(T)) Method. Methods. 2001 Dec;25(4):402-8. PubMed PMID: 11846609.

4. Wright WE, Shay JW, Piatyszek MA. Modifications of a telomeric repeat amplification protocol (TRAP) result in increased reliability, linearity and sensitivity. Nucleic Acids Res. 1995 Sep 25;23(18):3794-5. PubMed PMID: 7479015. Pubmed Central PMCID: PMC307284.

5. Machado-Pinilla R, Sanchez-Perez I, Murguia JR, Sastre L, Perona R. A dyskerin motif reactivates telomerase activity in X-linked dyskeratosis congenita and in telomerase-deficient human cells. Blood. 2008 Mar 1;111(5):2606-14. PubMed PMID: 18057229.

6. Cawthon RM. Telomere measurement by quantitative PCR. Nucleic Acids Res. 2002 May 15;30(10):e47. PubMed PMID: 12000852. Pubmed Central PMCID: PMC115301.
