# Supplementary material for: Generation of dyskeratosis congenita-like hematopoietic stem cells through the stable inhibition of DKC1
Source: Stem Cell Res Ther. 2021 Jan 29;12:92. doi: 10.1186/s13287-021-02145-8 (PMC7844988; doi:10.1186/s13287-021-02145-8)
Supplement: Supplementary file 2 — Additional file 2: Supplementary Figure 1. A) DKC1 expression after transduction with a library of DKC1-specific shRNAs, n=3. Analyses were performed 5-8 days post-transduction. B) Analysis of the vector copy number per cell (VCN/cell) after transduction with shRNA-LVs. Analyses were performed at least after 15 days post-transduction. Supplementary Figure 2. Gating strategy and analysis of Annexin V-based apoptosis assay. Representative dot-plots of flow cytometry analyses in DKC1-interferred CD34+ cells are shown. Supplementary Figure 3. Levels of CD34+ cells after 10 days in culture (n=3). Supplementary Figure 4. Telomere length in human CD34+ samples transduced with DKC1-shRNAs and scrambled-shRNA LVs and cultured for 8 days as described in Materials and Methods section. For comparison, also human peripheral blood cells and fresh CD34+ cells were analyzed (n=1). Supplementary Figure 5. Analysis of the clonogenic potential, discriminating between GM-CFU (A) and E-BFU (B) colonies, of CD34+ cells transduced with DKC1-shRNA LVs and scrambled shRNA-LVs (n=8). Data are expressed as mean ± SEM. Asterisks indicate significant differences determined by Student’s t test (*p < 0.05, **p < 0.01, ***p < 0.001, ****p < 0.0001). Supplementary Figure 6. Gating strategy and analysis of the NSG mice repopulating potential of CD34+ cells transduced with iDKC1 and scrambled-shRNA LVs. Representative dot-plots of flow cytometry analyses performed in the bone marrow samples from two mice transplanted with scrambled-shRNA LVs are shown. Supplementary Figure 7. Analysis of the VCNs in the bone marrow of recipient NSG mice at 3 months post-transplantation. NSG mice were transplanted with scrambled transduced CD34+ cells (orange dots, n=14) or iDKC1 interfered CD34+ cells (blue dots, n=8) [file 13287_2021_2145_MOESM2_ESM.pdf]

**shRNA**	**mRNA target**	**shRNA sequence**	**Position in *DKC1* gene (bp)**
**iDKC1**	*DKC1*; Exon 15	*GCUCAGUGAAAUGCUGUAGAA*	16756-16776
**iDKC2**	*DKC1*; Exon 15	*AUGUUACCUGGUGUGGUCAU*	16517-16536
**iDKC3**	*DKC1*; Exon 4	*CACUAUACACCUCUUGCAUGU*	5439-5459
**iDKC4**	*DKC1*; Exon 6	*CGGCUGCACAAUGCUAUUGAA*	6522-6542
**iDKC5**	*DKC1*; Exon 8	*GCACCUACAUUCGGACAUUAU*	7831-7851
**iDKC6**	*DKC1*; Exon 2	*CGGAAGUCAUUGCCAGAAGAA*	4439-4459
**iDKC7**	*DKC1*; Exon 3	*CGCUGAAGAAUUUCUUAUCAA*	4957-4977

Supplementary Table 1. Overview of the information about the shRNA sequences.

A

Condition	*DKC1* Average Ct	*GAPDH* Average Ct	ΔCt *DKC1 - GAPDH*	ΔΔCt (ΔCt iDKC - ΔCt Scrambled)	Fold difference in *DKC1* relative to Scrambled
**Scrambled**	28.54 ± 0.48	23.89 ± 0.67	4.68 ± 0.66	0.00 ± 0.66	1.00 (0.63-1.58)
**iDKC1**	30.11 ± 0.72	23.45 ± 0.62	6.66 ± 0.93	1.98 ± 0.93	0.25 (0.13-0.48)
**iDKC4**	32.36 ± 0.61	26.56 ± 0.57	6.16 ± 0.46	1.49 ± 0.45	0.36 (0.26-0.49)
**iDKC7**	28.16 ± 0.63	22.14 ± 0.68	5.63 ± 0.83	0.95 ± 0.83	0.52 (0.29-0.92)

B

Condition	*TERC* Average Ct	*GAPDH* Average Ct	ΔCt *TERC - GAPDH*	ΔΔCt (ΔCt iDKC - ΔCt Scrambled)	Fold difference in *TERC* relative to Scrambled
**Scrambled**	29.38 ± 0.60	24.73 ± 0.38	5.50 ± 0.53	0.00 ± 0.53	1.00 (0.69-1.44)
**iDKC1**	31.10 ± 1.05	24.42 ± 0.63	8.02 ± 0.52	2.52 ± 0.52	0.17 (0.12-0.25)
**iDKC4**	33.09 ± 1.18	26.62 ± 0.62	7.70 ± 0.61	2.20 ± 0.61	0.22 (0.14-0.33)
**iDKC7**	32.83 ± 0.67	23.76 ± 0.70	9.41 ± 0.65	3.91 ± 0.65	0.07 (0.04-0.10)

C

Condition	*CDKN1A* Average Ct	*β-actin* Average Ct	ΔCt *CDKN1A - β-actin*	ΔΔCt (ΔCt iDKC - ΔCt Scrambled)	Fold difference in *CDKN1A* relative to Scrambled
**Scrambled**	28.44 ± 0.78	23.90 ± 0.63	4.54 ± 0.34	0.00 ± 0.34	1.00 (0.79-1.27)
**iDKC1**	27.78 ± 0.96	24.00 ± 1.16	3.78 ± 0.28	-0.76 ± 0.28	1.70 (1.39-2.06)
**iDKC4**	29.05 ± 1.16	25.49 ± 0.89	3.56 ± 0.31	-0.98 ± 0.31	1.97 (1.59-2.44)
**iDKC7**	27.80 ± 0.76	22.53 ± 0.58	5.27 ± 0.36	0.72 ± 0.36	0.61 (0.47-0.78)

Supplementary Table 2. Compilation of RT-qPCR data in CD34+ transduced with scrambled and *DKC1*-shRNA LVs**. A**) Results of *DKC1* expression (n = 7). B) *TERC* expression data (n = 7). **C**) Expression of *CDKN1A* gene (n = 6). Triplicates were performed in every experiment and results are expressed as mean ± SEM.

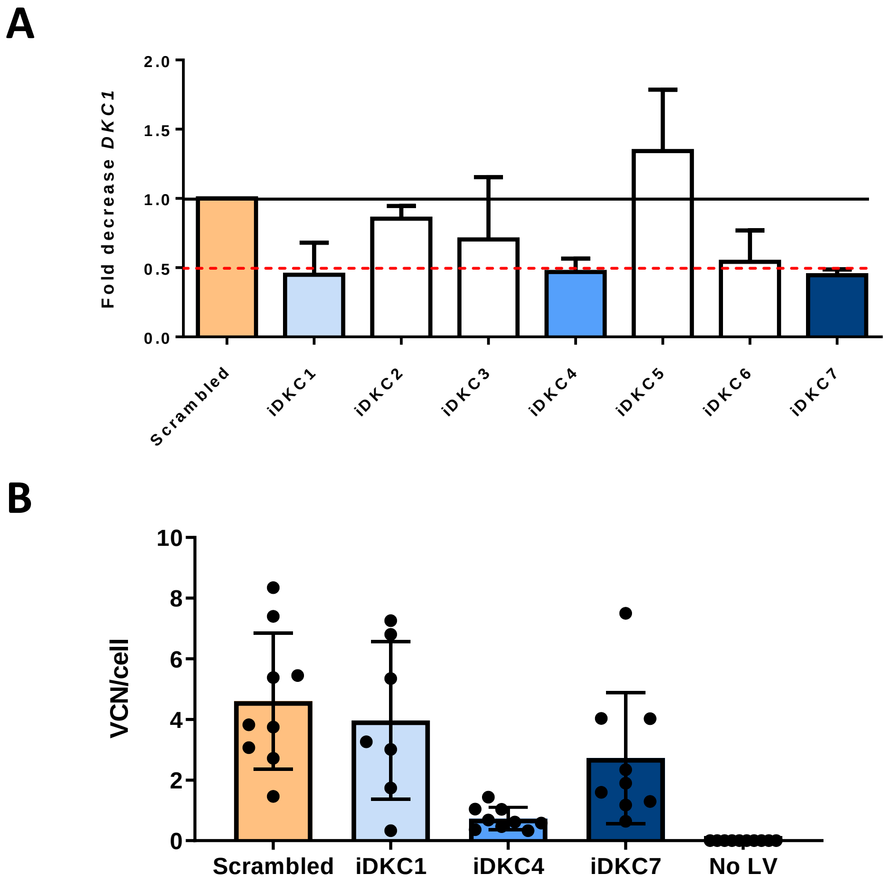

Supplementary Figure 1. A) *DKC1* expression after transduction with a library of *DKC1*-specific shRNAs, n=3. Analyses were performed 5-8 days post-transduction. **B)** Analysis of the vector copy number per cell (VCN/cell) after transduction with shRNA-LVs. Analyses were performed at least after 15 days post-transduction.

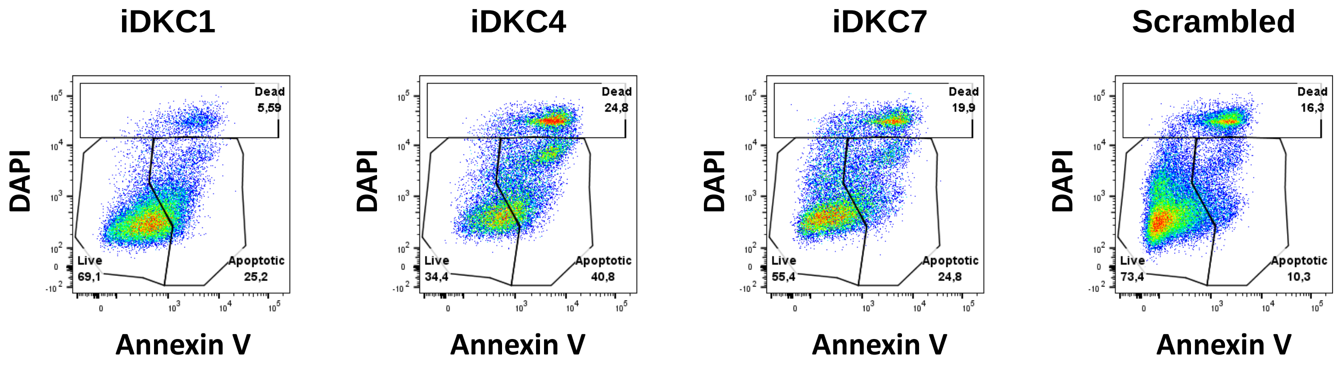

Supplementary Figure 2. Gating strategy and analysis of Annexin V-based apoptosis assay. Representative dot-plots of flow cytometry analyses in *DKC1*-interferred $CD34^{+}$ cells are shown.

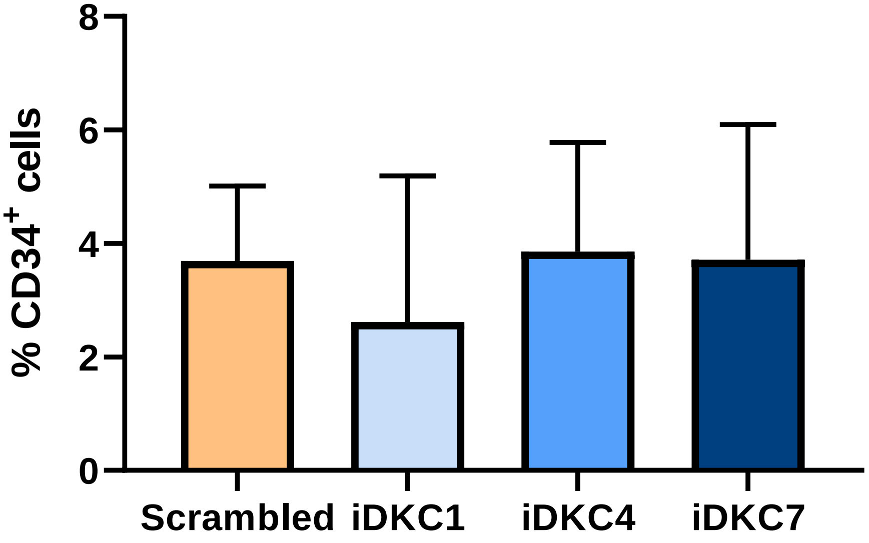

Supplementary Figure 3. Levels of $CD34^{+}$ cells after 10 days in culture (n=3).

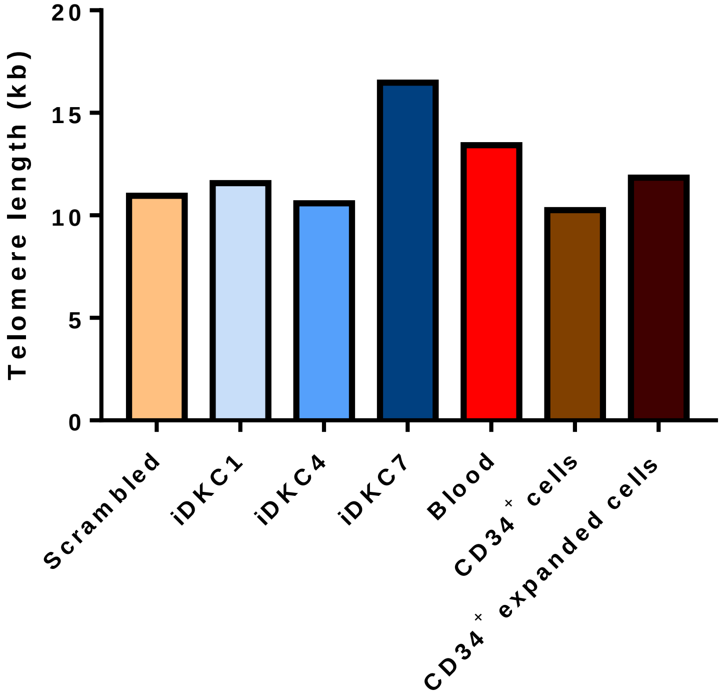

Supplementary Figure 4. Telomere length in human CD34$^+$ samples transduced with *DKC1-shRNAs* and scrambled-shRNA LVs and cultured for 8 days as described in Materials and Methods section. For comparison, also human peripheral blood cells and fresh CD34$^+$ cells were analyzed (n=1) .

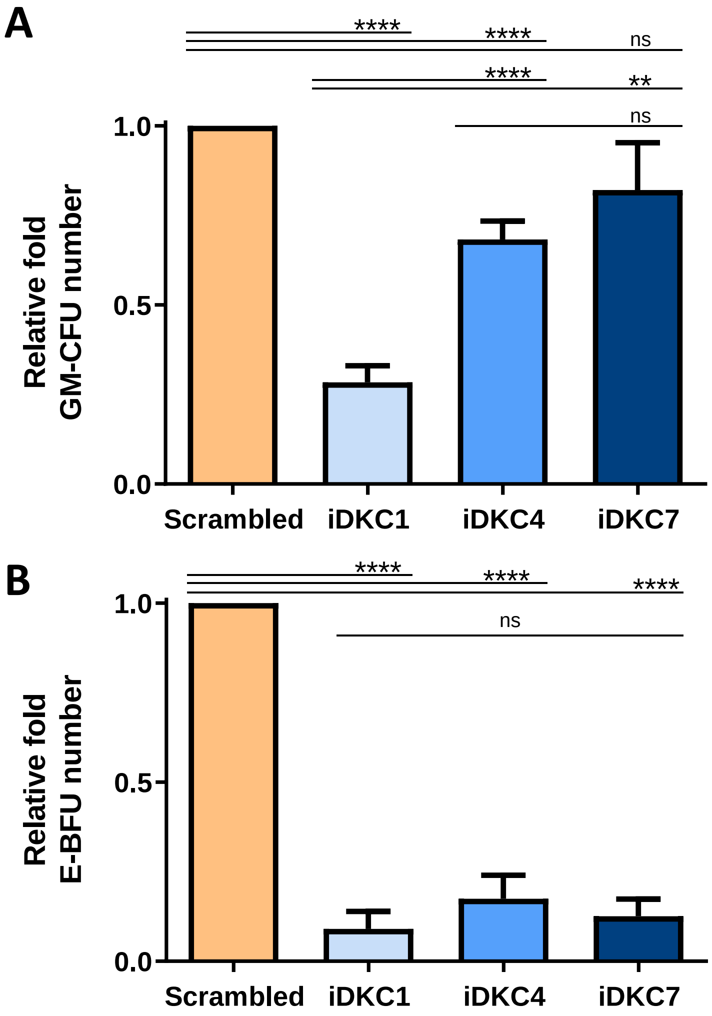

Supplementary Figure 5. Analysis of the clonogenic potential, discriminating between GM-CFU (A) and E-BFU (B) colonies, of $CD34^+$ cells transduced with *DKC1*-shRNA LVs and scrambled shRNA-LVs (n=8). Data are expressed as mean ± SEM. Asterisks indicate significant differences determined by Student's t test (*p < 0.05, **p < 0.01, ***p < 0.001, ****p < 0.0001).

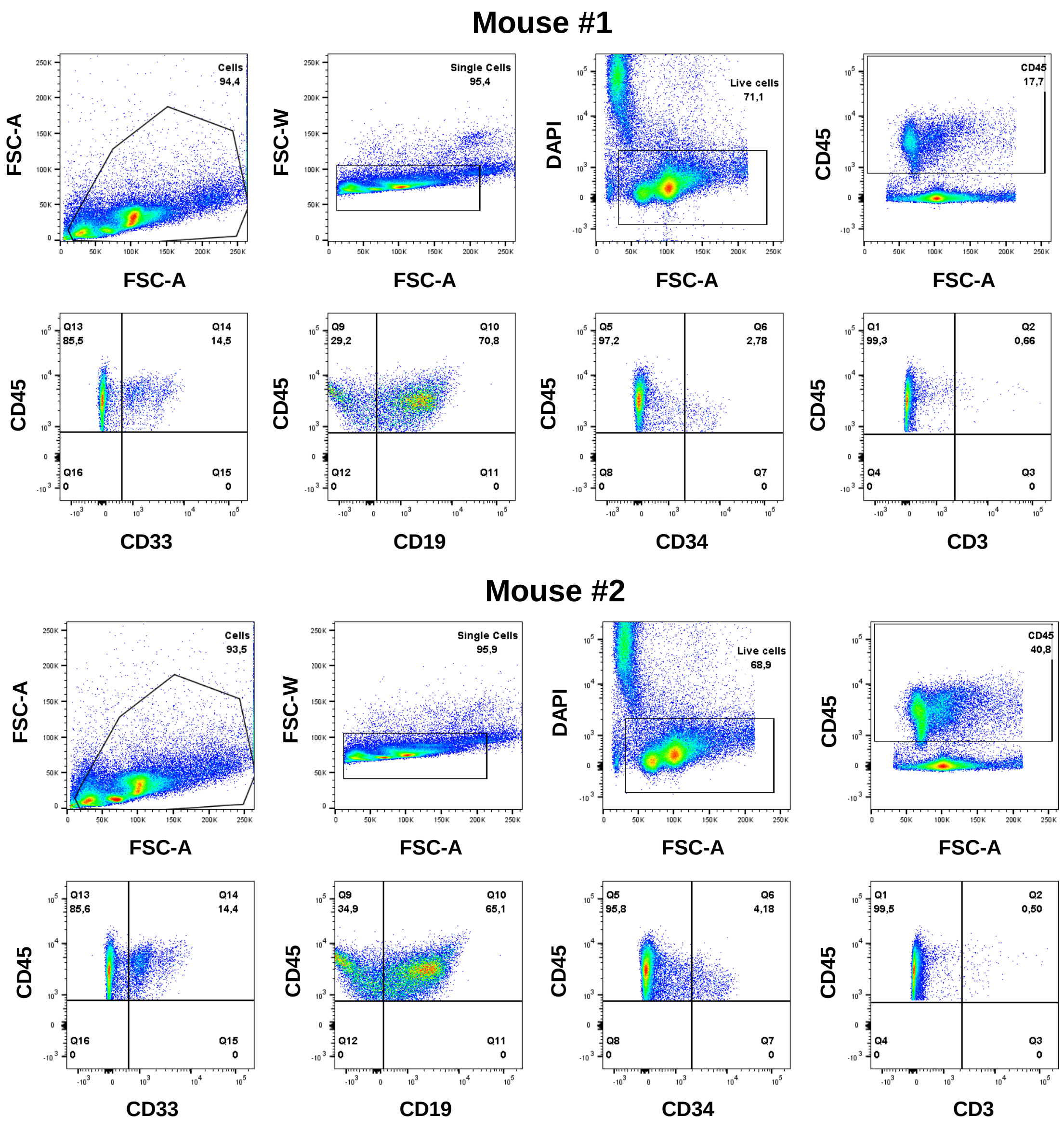

Supplementary Figure 6. Gating strategy and analysis of the NSG mice repopulating potential of CD34$^+$ cells transduced with iDKC1 and scrambled-shRNA LVs. Representative dot-plots of flow cytometry analyses performed in the bone marrow samples from two mice transplanted with scrambled-shRNA LVs are shown.

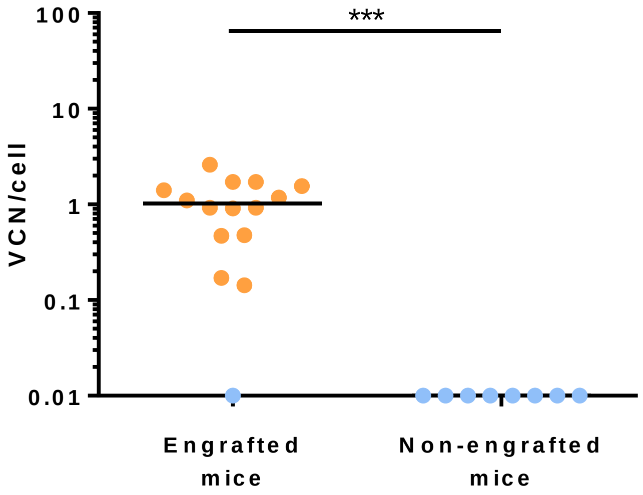

Supplementary Figure 7. Analysis of the VCNs in the bone marrow of recipient NSG mice at 3 months post-transplantation. NSG mice were transplanted with scrambled transduced CD34^{+} cells (orange dots, n=14) or iDKC1 interfered CD34^{+} cells (blue dots, n=8).
